# Progress of Research on Urban Growth Boundary and Its Implications in Chinese Studies Based on Bibliometric Analysis

**DOI:** 10.3390/ijerph192416644

**Published:** 2022-12-11

**Authors:** Xiaoyang Liu, Weihao Shi, Sen Zhang

**Affiliations:** 1 School of Urban Design, Wuhan University, Wuhan 430072, China; 2 Research Center for Hubei Habitat Environmental Engineering & Technology, Wuhan 430072, China; 3 Shenzhen Research Institute, Wuhan University, Shenzhen 518057, China; 4 School of Architecture, Tianjin University, Tianjin 300072, China

**Keywords:** urban growth boundary (UGB), ecological protection, bibliometric analysis, development and evolution, delimitation means, implementation management, China

## Abstract

Urban sprawl is a development theme of cities all over the world, especially in developing countries with rapid urbanization, and the long-established rough and outward urban growth pattern has brought about a series of social and ecological problems. As an important tool in controlling urban sprawl in western countries, the urban growth boundary (UGB) has become one of the three major policy tools in the national spatial planning system since it was introduced into China. Combined with a bibliometric analysis, this literature review summarizes UGB studies on development and evolution, delimitation means, and implementation management and provides references for studying UGB adaptability in China. The results show that: (1) Originating from Howard’s garden city concept, UGB studies have formed a relatively complete system of “theoretical basis, technical methods, supporting policies, and implementation management” through long-term empirical research in foreign countries. With a relatively late start in China, UGB research currently focuses on different situations between China and abroad and the adaptation of China’s localization. (2) UGB delimitation mainly includes two aspects: forward expansion, which, from the urban development perspective, is mainly supported by cellular automata (CA) urban growth simulation; and reverse restriction, which, from the ecological protection perspective, is supported by ecological security pattern construction, ecological sensitivity evaluation, and land suitability evaluation. (3) Many foreign UGB implementations have different forms and more flexible and comprehensive corresponding supporting policies. However, the current state of research in China in this area is still insufficient. Against the background of the national spatial planning system reform, the findings of this review provide references for delineating UGB that considers ecological protection and urban development under the scenarios of planning, formulating a supporting mechanism for multi-subject participation and multi-party coordination, and establishing an adjustment system based on implementation effect evaluation.

## 1. Introduction

Urbanization will remain one of the major trends in the transformative development of human society in the 21st century [1]. Many developing countries are currently experiencing a sustained wave of urbanization [2]. In the 40 years since China’s reform and opening up, the urbanization rate has increased from 17.9% in 1978 to 64.7% in 2021. Along with large-scale urban expansion, the built-up area has expanded eight times, and the speed of land urbanization far exceeds the speed of population urbanization [3]. The urban development model based on rough and outward expansion has led to a series of practical problems, such as the disorderly sprawl of built-up areas, inefficient use of land resources, and the impairment of ecosystem service functions [4,5,6,7,8]. Facing the above-mentioned problems, the Central Urbanization Conference, the Fifth Plenary Session of the 18th Central Committee, the Fifth Plenary Session of the 19th Central Committee, and other important conferences have successively put forward the strategies of “controlling urban sprawl”, “scientifically constructing urbanization pattern”, and “optimizing spatial development pattern and national land protection”, which show that it is urgent to actively explore the transformation of urban development strategies. On the other hand, most of the space management work that China has carried out has not achieved the intended effect. Previous multiple control lines have problems such as overlapping scope, management conflict, and frequent adjustment. Therefore, controlling the urban scale and optimizing the spatial pattern have become the focus of China’s urban growth management in the new era.

Urban sprawl was initially dominated by American cities. Due to its many adverse consequences, the United States and other developed countries adopted a series of urban growth management tools to deal with urban sprawl and achieved certain success [9,10]. Among them, Urban Growth Boundary (UGB), as a representative policy tool, has attracted continuous attention. Its original concept was “the boundary between urban and rural land”, which has attracted continuous attention as a representative tool for urban growth management and has played a positive role in controlling urban sprawl, achieving smart urban growth and optimizing the ecological environment [11]. However, since China’s rapid urbanization in the 1990s, many facts have shown that Chinese cities are repeating the same mistakes of American urban sprawl decades ago [12]. In China, the urban growth boundary is an artificial boundary between construction and non-construction land set by urban planning authorities to guide rational urban development and protect ecological resources. In 2006, the term “urban spatial growth boundary” appeared for the first time in the “compilation method of urban planning implementation” of China’s Ministry of Construction. In 2014, the Ministry of Housing and Construction and the Ministry of Land jointly identified 14 cities nationwide to carry out pilot work on delineating urban development boundaries. In 2017, the report of the 19th National Congress proposed to “complete the delineation of the three control lines of ecological protection red line, permanent basic agricultural land, and urban development boundary”, renewing the focus of the urban development boundary.

In fact, although UGBs have been widely used internationally, the rich forms derived from this policy tool in specific local practices have made it impossible to have a unified conceptual definition and a technical process so far. In addition, owing to the differences in national conditions, UGB has been in the localization adaptation stage since its introduction in China. Under the current circumstances, such as frequent revision of national and local norms and guidelines, non-unified technical processes, and weak supporting management system, it is of great significance to summarize the research progress of UGB and propose its future application in China. This study first applies bibliometric methods to quantify the current status of UGB research at home and abroad, then summarizes the development of UGBs, as well as their demarcation methods and supporting policies, and finally provides a general review and proposes suggestions for future development in China.

## 2. Methodology

With the continuous innovation of computer technology and the wide application of big data analysis software in the field of scientific research, the bibliometric method is a scientific analysis method that is developing vigorously. CiteSpace is one of the representative bibliometrics software tools, which can perform text analysis, data mining, and visualization operations on a literature database. By combining social network analysis, cluster analysis, and co-citation analysis, it can intuitively display the knowledge structure and topic evolution of a specific discipline, demonstrate the footprints of scientific development, and track its research hotspots, research frontiers, and research trends [13].

**Figure 1:**
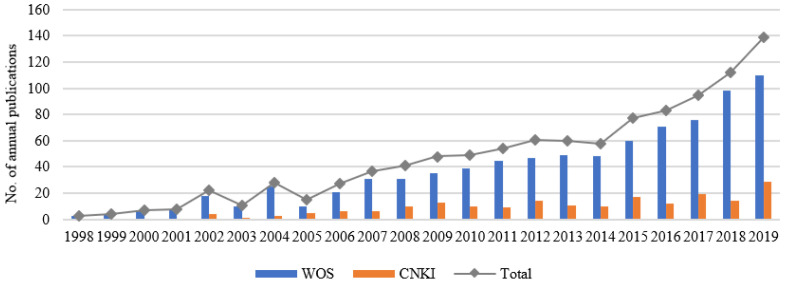
Number of annual publications on urban growth boundary studies from 1998 to 2019. WOS, Web of Science; CNKI, China National Knowledge Infrastructure.

We conducted a literature review based on the databases of Web of Science and China National Knowledge Infrastructure (CNKI), using the search terms “urban growth boundary” and “urban development boundary”. After rectifying, screening, and sorting the search results, we selected 845 and 188 pieces of literature from the Web of Science and CNKI databases, respectively (Figure 1). As a result, a total of 1033 Chinese and foreign literature texts on UGBs were obtained and analyzed quantitatively using CiteSpace.

## 3. Results

### 3.1. Bibliometric Analysis of UGB

Figure 1 shows that during 1990 and 2019: (1) The total number of domestic and foreign publications has generally shown an upward trend, with a significant upward trend from 2014 onwards, the reason for which should be related to China’s decision to launch a pilot project to delineate UGBs in 2014. (2) International research on UGBs began in 1998, and in China, it began in 2002 with a paper entitled “Urban growth Management in the United States” by Zhang Jin [14]. (3) Since the beginning of the study, the amount of Chinese literature has always been less than that of foreign literature.

#### 3.1.1. Research Hotspots of UGB

**Figure 2:**
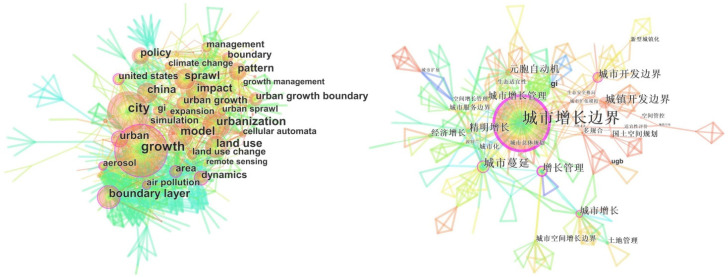
Co-occurrence map of high-frequency keywords in foreign languages (**left**) and Chinese (**right**) for urban growth boundary research, 1998–2019. (Please refer to Appendix A Table A1 for the translation of Chinese words).

**Figure 3:**
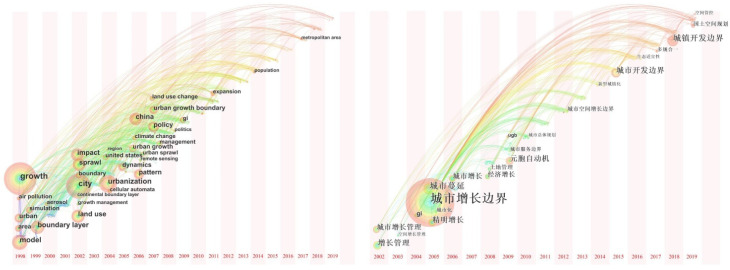
Time-zone maps of the evolution of high-frequency keywords in foreign languages (**left**) and Chinese (**right**) for the study of urban growth boundaries from 1998 to 2019. (Please refer to Appendix A Table A1 for the translation of Chinese words).

Focusing on international research, it can be summarized from the co-occurrence network of high-frequency keywords (Figure 2) that “growth”, “urbanization”, “model”, “land use”, “sprawl”, “policy”, “cellular automata”, etc., are the most frequent keywords in terms of research content; “city” and “metropolitan area” in terms of research scales; and “United States” and “China” in terms of research objects. The time-zone diagram of the dynamic evolution of high-frequency keywords (Figure 3) can be summarized as follows: From the initial stage (1998–2002), international attention was paid to the urban sprawl control research based on the prevention and control of air pollution and other environmental damage. Towards the middle stage (2002–2006), land use simulation research was combined with CA, and then developments led to a focus on the improvement of dynamic monitoring and policy systems combined with remote sensing (RS) and geographic information systems (GIS) after 2006. In terms of domestic research, the following keywords appeared more frequently: urban sprawl, growth management, metacellular automata, smart growth, green basic service facilities, and territorial spatial planning (Figure 2). According to Figure 3, UGBs in China have undergone development processes of “urban growth boundary (2005), urban development boundary (2015), and cities and towns development boundary (2018)”. In the early stage (before 2005), international experience was introduced and summarized. The middle stage (2006–2014) focused, on the one hand, on urban expansion simulation based on CA, and on the other hand, this was combined with urban master planning. However, this area of study was not widely promoted until 14 pilot projects of urban development boundary demarcation were proposed. Finally, from 2015 to the present, urban development boundaries have been proposed under the background of new urbanization, multi-planning, and changes in territorial spatial planning.

In general, UGB research in China has gradually evolved from the initial conceptual connotation analysis and summary of foreign experiences to research on boundary delineation methodologies and practices. Among the journal papers on the topic of “urban growth boundary” searched in CNKI since 2014, more than 60% are on the technical methodology of the boundary and can be roughly divided into three categories: The first category includes summaries of the pilot research work of urban development boundary in China [15], among which the representative cases of pilot cities, such as Shanghai, Hangzhou, and Wuhan, have also summarized their respective technical method systems. The second category includes discussions of how to use new technologies and tools for boundary delineation, such as ecological safety patterns, green infrastructure, and ecological suitability assessment, as the basis for the bottom-line development limit [16], or CA model-based spatial expansion simulation as the basis for urban growth prediction [17]. The third category focuses on the discussion of the methods of urban development boundary delineation under the context of territorial spatial planning reform, such as the “integration of multiple regulations” and “dual evaluation” [18].

#### 3.1.2. Research Frontiers of UGB

**Figure 4:**
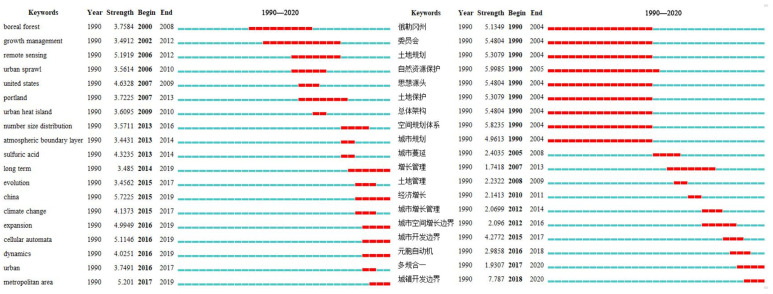
Keyword emergence diagram of urban growth boundary research in foreign languages (**left**) and Chinese (**right**). (Please refer to Appendix A Table A1 for the translation of Chinese words).

In terms of international research, the keyword emergence map (Figure 4) can be summarized as follows, the red marks represent when the keywords appear and end. In the first decade of the 21st century, “boreal forest”, “growth management”, “remote sensing”, and “Portland” were the phase research hotspots. From 2010 onwards, a number of research hotspots emerged, and more than half of them have been maintained to date, including “long-term planning”, “China”, and “cellular automata”. This indicates that before 2010, UGB did not attract widespread attention in China and was studied on behalf of the United States (US), whereas after 2010, especially after the year 2014, a large number of studies related to concept’s introduction, spatial modelling, and the practical implementation of UGB in China have been conducted, which have had a greater impact internationally. As for domestic research, it can be summarized from Figure 4 that before 2004, domestic scholars focused on summarizing and sorting out foreign experiences, laying the foundation for the localized application of UGBs in China. However, after 2005, the boundary delimitation methods and technical means have been explored, but without the formation of a unified paradigm. Therefore, the emergent words include several definitions, such as “urban spatial growth boundary”, “urban development boundary”, and “cities and towns development boundary”. The concept of sexuality, the simulation of urban spatial expansion based on CA, emerges in large numbers in the short term.

Next, based on the above research concerns at home and abroad, this study elaborates the development process, demarcation method, and supporting policies of UGB.

### 3.2. Literature Review of UGB

#### 3.2.1. Development Process of UGB

UGBs were first proposed in Western countries in response to the negative effects of urban sprawl [19]. The origins of the idea can be traced back to Howard’s garden city theory in 1898 and the Green Belt in the Greater London Plan of 1944 [20]. The garden city theory proposed the construction of ecologically protected green belts on the periphery of urban centers to control urban sprawl, and the permanent ‘Green Belt’ was then used as a limit in the Greater London Plan. In 1976, the city of Salem formally put forward the UGB theory for the first time. In the 1970s, the US states of Oregon and Washington specified the UGB as a mandatory part of urban spatial planning [21], after which the UGB was widely promoted internationally.

The practical studies related to UGB can be divided into three types. The first is the green belt policy represented by the Greater London Plan in the UK, and similar cases include the green belt plan in Seoul, Korea, the green heart in Randstad, the Netherlands, and the circular green corridor in Paris, France. The central idea is that urban sprawl can be limited through the design of green public spaces. The second type is the smart growth policy, with American cities as representative cases, such as Portland in the US and Melbourne in Australia. The main goal here is to guide the intensive and efficient development of cities and protect the ecological environment. The third type is the urban zoning control policy, with Japan as a representative case, which controls urban sprawl by designating different functional zones (such as urbanization promotion zones and urbanization control zones). To sum up, the nascent period of UGB research started with the early green belt policy [22]; the development period was represented by the smart growth theory [20,23]; and the mature period occurred after 2000, with the emergence of deeper and broader theoretical research on boundary modeling, boundary management, and boundary form optimization [24,25].

**Figure 5:**
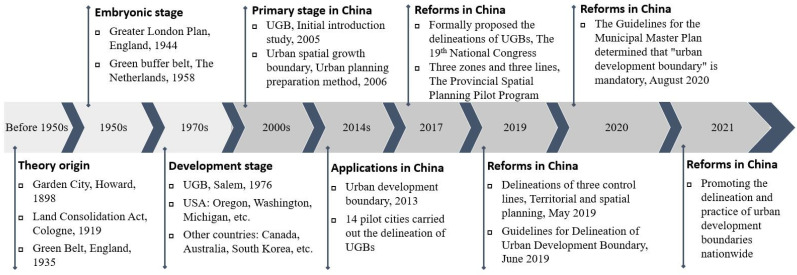
Development of theories and applications of urban growth boundary.

The initial research on this urban growth management tool began in the late 1990s, mainly through the introduction of the theories and practices of growth management in the US, and then, considering China’s national conditions, scholars gradually shifted their focus to the localized application of UGBs. In China, the evolution of the UGB since its formal introduction can be roughly divided into three stages. (1) The “urban spatial growth boundary” stage (2006–2012) is the first. In 2006, the “Urban Planning Preparation Methods” proposed that the “urban spatial growth boundary” should be defined in the urban masterplan. However, no systematic regulations in the practice of the masterplan were formed in the following years. (2) The stage of “urban development boundary” (2013–2016) came next. In 2013, the Central Urbanization Conference proposed to “delineate the development boundary of mega-cities as soon as possible” and then launched 14 pilot projects on urban development boundary delineation nationwide in July 2014, marking a transformation from theoretical research to concrete practice, with the goal of “strengthening spatial control”. (3) The stage of “cities and towns development boundary” (2017–present) is the last stage. In 2019, the “Guide to the Delineation of Urban Development Boundaries (for trial implementation)” defined the basic concept of urban development boundary, and in 2020, the “Guide to the Preparation of Municipal Territorial Spatial Master Plans (for trial implementation)” made urban development boundaries mandatory. Under the guidance of the idea of “strictly controlling the increase and revitalizing the stock”, the UGB has gradually developed from the initial focus on controlling urban sprawl and protecting arable land to the direction of promoting the transformation of urban development and shaping a sustainable and beautiful national space [26]. Figure 5 shows the evolution of theoretical and practical research on UGBs in China and abroad.

#### 3.2.2. Delineation Method of UGB

Since the concept of UGB was introduced, researchers have explored several methods of boundary delineation, but a unified methodology system has not yet been formed. Based on years of theoretical and practical experience, boundary delineation methods can be categorized as qualitative and quantitative, of which the latter are represented by the forward expansion and reverse restriction methods.

##### Qualitative Delineation Method

The Frey and Portland methods are the most representative qualitative approaches. The specific process of Frey’s qualitative delineation method includes “development problem identification, relevant data collection, growth scale prediction, and growth boundary delineation”. The main idea is to predict the future land area of a city based on its current population size, development and construction costs, supporting infrastructure, etc. The Portland qualitative delineation method first sets four possible development scenarios for a city’s future development, with each scenario corresponding to an urban spatial evolution characteristic, and selects the optimal solution by comparing multiple factors such as land construction and development intensity, average traffic travel distance, and green space system structure to form a preliminary UGB. The final UGB is then obtained based on the current land use situation, distribution of ecologically sensitive and fragile areas and public service facilities, and evaluation of construction land suitability.

##### Quantitative Delineation Method

**Figure 6:**
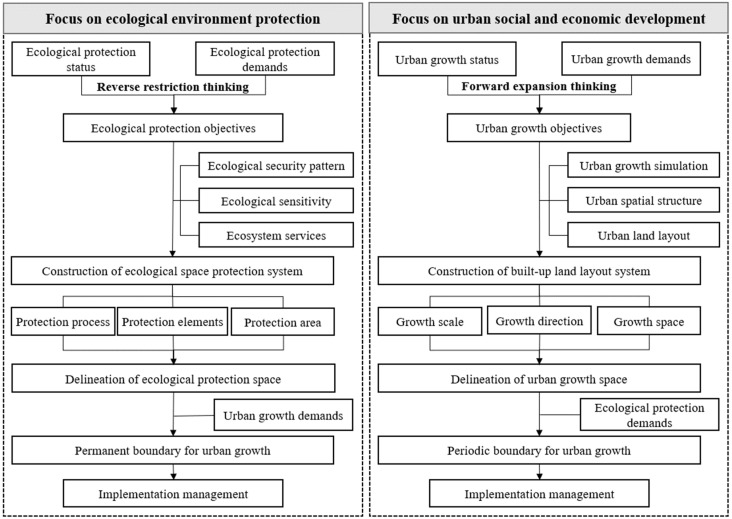
“Reverse limiting thinking” (**left**) and “Forward expansion thinking” (**right**) delineate the technical process of urban growth boundary.

In terms of quantitative boundary delineation, the traditional method in China is to use a 20-year planning period as the basis for calculating the expected land scale in a given year based on the population size and per capita construction land, and then combine it with the evaluation of construction land suitability and resource- and environment-bearing capacity and subjective experience to lay out the urban space and finally determine the urban construction land boundary. However, in the context of the new-type urbanization, the population size is constantly changing dynamically under the constraints of natural resources, and the per capita construction land index is increasingly different in different regions. The construction land boundary demarcated by the above traditional methods is often breached, and thus a more scientific and objective demarcation method has been under exploration. In summary, the quantitative delineation method mainly adopts two models, urban development-oriented and ecological protection-oriented (Figure 6).

(1) Forward expansion method based on urban development orientation

The forward expansion method is focused on urban development needs and specifies the scale of future land use according to the direction of development and construction, the scale of population growth, etc. It is an additive theory, including the static meritocratic method and the dynamic simulation method. The former is to estimate the value of land development by evaluating the suitability of construction land and the coverage of public service facilities, etc. and to select eligible sites as internal sites with developable boundaries by integrating expert experience. The latter is based on various types of urban expansion models, combining natural, social, and economic drivers to simulate urban land expansion, of which metacellular automaton (CA) is the most widely used model, which can be combined with GIS and RS to simply abstract complex real spatial patterns, and metacellular transformation rules can be set up to depict the changes of metacells under the combined influence of various factors (e.g., neighborhood factors, own state, etc.) in the next phase, and finally, the rough urban spatial pattern is obtained [27,28]. However, the traditional CA model has shortcomings in capturing the macro socio-economic drivers of urban growth, in particular, the human decision-making process is not incorporated into the model, so that it cannot reflect the human–land relationship behind land use change.

With the improvements in big data acquisition and RS technology, some spatially relevant drivers, such as planning policies and ecological reserve settings, were introduced into CA models. During the process, many statistical methods (such as logistic regression analysis and subjective–objective assignment methods) were used to quantify and calculate the weights of each driver as one of the bases for model parameter adjustment. Meanwhile, an increasing number of artificial intelligence techniques, such as genetic, random forest, particle swarm-ant colony, neural network, and other machine learning algorithms, were used to optimize CA models [29,30,31,32]. In combination with statistical methods and artificial intelligence optimization techniques, many CA-improved models have been applied to urban growth simulation, such as constrained CA, SLEUTH, CLUE-S, Logistic-CA, CA-Markov, GeoSOS, and FLUS models [33,34,35,36,37,38]. The simulation results of these improved models are more scientific and accurate than those of traditional CA models. Long et al. used constrained CA to delineate the UGB of Beijing at the three levels of central city, new city, and township, and their simulation results showed significant differences with the UGB formulated in the Beijing master plan [33]. Liu et al. used different ecological security pattern scenarios as FLUS model constraints to conduct multi-scenario simulations of urban land development in the study area in 2035 and found that strengthening ecological protection could effectively control urban spatial sprawl [39]. Firozjaei et al. used global and directional approaches to optimize the accuracy of the CA–Markov simulation and tested urban sprawl simulation results using Pilka square statistics, Shannon entropy, and the superiority index, which showed that the accuracy of the optimized CA–Markov model was better than that of the traditional model [40]. Liang et al. combined a top-down regional planning policy scenario with a bottom-up FLUS model and demonstrated that the FLUS model with coupled system dynamics and CA models could be used to simulate urban growth simulation under the influence of different driving factors [38].

(2) Reverse qualification method based on ecological conservation orientation

Compared with the additive thinking of the forward expansion method, the reverse restriction method embodies subtractive thinking, which is based on the “anti-planning” theory. The main research methods include ecological network construction, green infrastructure analysis, ecological security pattern evaluation, ecological sensitivity analysis, and ecological suitability evaluation [41,42,43,44]. Zhou et al. predicted the land use demand based on the ecosystem service value maximization optimization model and designed four future land-use change scenarios of natural development, ecological security, multi-regulation integrity, and ecological health by building an ecologically restricted area [39]. Jiang et al. selected 10 indicators from the three aspects of natural endowment, location conditions, and ecological environment to construct an ecological suitability model to evaluate the spatial pattern level of land development and construction in the study area. Their results provided a basis for analyzing the system of synergistic development strategies between development and conservation in the development and construction of land on low hills and gentle slopes [44]. Fu et al. constructed two spatial development scenarios for towns based on ecological security patterns through hotspot analysis and a multi-objective decision-making method and proposed a functional and structured ecological spatial layout framework based on the results [45]. From a comprehensive perspective, this approach was similar to the logic of the “three zones and four lines” spatial control used in China’s urban planning system. In China’s pilot urban development boundary delineation practice, the reverse-limiting method has been widely adopted to coordinate the overall urban and land use planning to determine the final boundary shape.

However, it should be noted that the forward expansion method starts from the concern for urban development needs, whereas the static merit method determines the boundary between construction and non-construction land through various evaluation methods, excluding the ecological resource background. The dynamic simulation method is essentially based on the premise of satisfying the land use scale under the population growth scenario, and its simulation, which predicts the scope of land use, still has the possibility of encroaching on the surrounding ecological resources. Although the inverse constraint method prioritizes the delineation of various constraints and specifies the spatial extent of key ecological resources that need to be protected, the boundaries projected by this method are equivalent to the static boundaries of the final form of urban development that cannot be breached, allowing the boundaries that cannot play a phased role in guiding the dynamic development of urban growth to be too large.

#### 3.2.3. Supporting Policies of UGB

The continuous practice of UGB has instigated research on its supporting policies in the implementation process. In Europe and the US, the UGB has been accompanied by a number of management systems, such as the designation of spatially protected suburban areas to identify the land that should be protected, the designation of priority development areas to identify the most suitable land for development, and the identification of development levels for different areas. It can be seen that the UGB is not just a technical boundary, but a policy toolbox composed of a series of factors [23,46,47,48,49,50]. In the US, the country with the longest history of urban growth management practice internationally, growth management can be roughly divided into four stages: the first stage (1969–1976), which focused on the formulation of environmental protection plans; the second stage (1977–1988), which focused on the coordination of the responsibilities of government departments at all levels; the third stage (1989–1997), which focused on the coordination of interest groups at multiple levels and the development of incentive mechanisms; and the fourth stage (1998–present), which focused on the development of a series of plans to improve ‘smart growth’. Growth management in the US has evolved in a holistic and diverse manner, from top-down to collaborative control, regulatory to incentive control, and local to regional control, with the related management tools gaining wider acceptance gradually. At the same time, complementary policies have been enacted to ensure the implementation of UGBs (Table 1), which can be used individually or in conjunction with UGB policies to ensure flexibility and comprehensiveness in the implementation of controls.

The study of China’s localized policy management complemented by the implementation of supporting boundary delineation on the ground is a major concern at this stage. For example, Yang comprehensively compared the theoretical and practical cases of UGBs in China and abroad in terms of development evolution, regulation setting, and social governance and proposed that China’s work on the delineation of the three control lines should emphasize the attributes of policy tools and improve the corresponding aspects of policy formulation, institutional design, and control instrument operation [51]. Xu explored the rigid elasticity of China’s urban development boundaries from the perspective of China’s current stage of territorial spatial planning reform [52]. Lin et al. summarized the achievements and experiences of 14 urban development boundary demarcation pilots, summarized the implementation management measures from the aspects of control measures, adjustment evaluation, supporting policies, etc., and put forward measures from the perspective of implementation and application, such as coordinating rigid control and flexible management, delimiting urban development boundaries across the whole region, and fully connecting the two control measures [15]. Yin et al. put forward three full-coverage systems for territorial space: a planning system, planning management and control requirements, and planning management means, including classified and differentiated management inside and outside the boundary and an exploration of local management system innovation [53]. Xie et al. proposed a city–county coordination path of “strategic leadership, index constraint, three lines coordination, and integrated monitoring” to promote the transformation of UGB management from static to dynamic, from quantitative to qualitative, and from rigid to flexible [18]. Huang et al. emphasized the adoption of a “double line” control model, that is, a permanent and phased boundary control time frame, to achieve the goals of both control and guidance [54]. However, as UGBs have been practiced in China for a relatively short period of time, with most of the existing studies focusing on the technical process of boundary delineation, there is still a lack of concrete operational experience in exploring UGBs at the management level, such as supporting policies for the boundaries.

## 4. Discussion

### 4.1. General Review of the Study

(1) The UGB theory is a policy tool proposed in the US to cope with urban sprawl. Early green belt planning was aimed at controlling the spatial spread of urban built-up areas, and with metropolitan development and population growth, its function gradually changed to reasonably guiding the orderly development of urban or regional spaces. The UGB theory was first proposed and practically implemented in Salem, USA in 1976, and then it was gradually adopted and emulated by other cities worldwide. Since its introduction into China, the UGB policy has become one of the three major policy tools of the territorial spatial planning system, with the aim of guiding rational land development and ecological resource protection around the city.
(2) Theoretical research on UGBs can be divided into three main directions: first, the definition of the UGB concept, the evolution of its connotation, and the comparison between China and foreign countries; second, the technical routes and methods of boundary delineation; and third, the supporting management and implementation policies of the boundary control system. The technical methods of boundary delineation are the focus of research, with the “forward expansion” and “reverse restriction” methods oriented toward the needs of urban development and ecological protection, respectively; both methods have their own advantages and shortcomings. However, the boundaries drawn by the reverse restriction method are too large in scope and cannot guide spatial expansion in an orderly manner; the forward expansion method can guide the direction of urban growth in a phased manner, but it does not effectively plan the spatial conjugation of ecological resources and construction land. In addition, owing to the limitations of the model, the development scale prediction, driving factor selection, model parameter tuning, and growth rule formulation are all difficulties of this method.
(3) In parallel with theoretical research, relevant practices are also being promoted. Since the introduction of the UGB concept, related planning practices, such as the green belt planning in London and Seoul, smart growth planning in Portland and Melbourne, and planning zoning policy in Japan, have been rapidly promoted. At the same time, most states in the US have enacted UGB regulations. There are few practical studies in China, and previous policy tools with similar UGB effects, such as Beijing’s restricted zone planning, Shenzhen’s basic ecological control line, and Chengdu’s ecological security pattern, have laid the foundation for the practical application of UGB. However, the Chinese government has identified 14 first pilot cities for boundary delineation in 2014 and subsequently expanded the pilot list to 600 cities across the country, and related practices are being explored and mainly focused on the urban scale.

In general, international countries have led to the establishment of a relatively sound theoretical foundation, technical methods, and implementation management system in long-term practical research. However, in China, the relevant research has a history of only just more than ten years, and it mainly collates foreign theoretical and practical studies for the localized application of UGB in China, focusing on the conceptual connotation, the differences between China and abroad, delineation methods, and other aspects of theoretical research, whereas practical studies on delineation standards, technical processes, and management systems have not yet formed a system.

### 4.2. Suggestions on the Localization Development of UGB in China

In the context of large-scale urbanization and national spatial planning system reform in China, the implementation of UGB policy is an inevitable choice. Thus, it is of great significance to guide China’s urban spatial development by borrowing experience from western countries. However, the UGB practice in the United States mainly advocates the protection of the ecological environment and promotes the vigorous and intensive development of urban central areas. Meanwhile, the main task in China is not only to balance land supply and demand inside and outside the boundary and to protect ecological resources such as water bodies and farmland, but also to guide rational urban development.

#### 4.2.1. Delineation of UGB Integrating Multi-Scenario Planning

**Figure 7:**
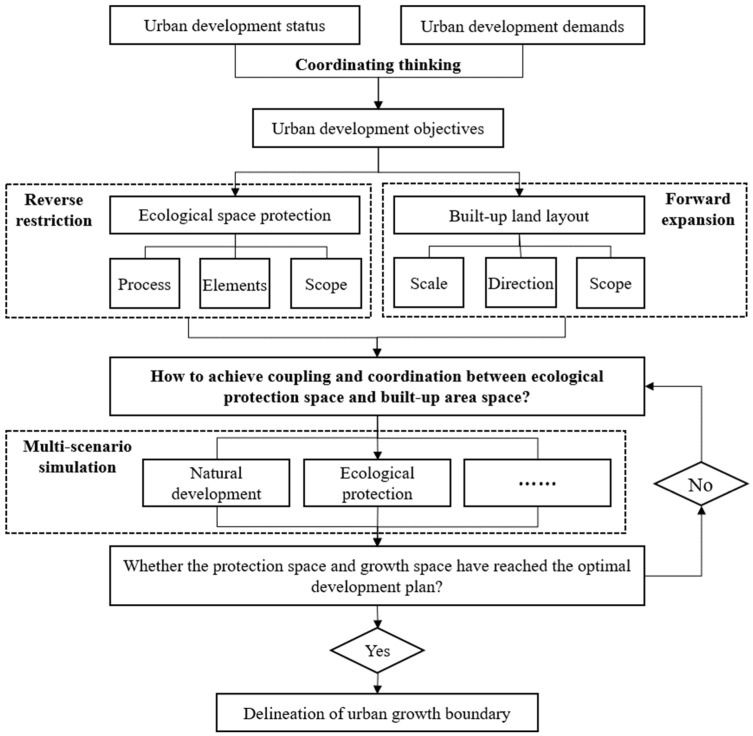
Research framework of urban growth boundary based on multi-scenario planning.

China’s territorial spatial planning is in a period of exploration and transition. In the field of practical application, the 14 pilot cities have mostly adopted the inverse constraint method to push back the boundary. Although from the prospective of theoretical research, both the inverse qualification and forward expansion methods have been extensively studied, the research on synergistic coupling of the two methods is still in its infancy. In the context of the rapid advancement of big data and computer technology, multi-channel access to data from multiple sources, combined with the extensive use of RS and GIS technologies and the continuous improvement of complex spatial dynamic simulation systems have provided the planning field with refined and scientific means for understanding urban development. It is proposed that the scenario planning method be introduced to design possible combinations of urban dynamic development under different constraints or facilitated by the perspective of conservation and development, which also responds to the demand for “multiple-scenario analysis for territorial spatial planning” in the preparation of territorial spatial planning, making urban spatial development more optional and scientific (Figure 7). Scenario planning generally consists of three steps: “Influencing factor identification, scenario condition presupposition, and spatial development simulation”. The identification of influencing factors is a combination of controlling and guiding factors to cope with uncertain development according to the natural resource endowment and the law of urban development in the study area. Scenario condition presupposition is used to construct different development scenarios through different combinations of hypothesis conditions, such as natural development, economic priority development, and ecological constraint development scenarios. Spatial development simulation is based on the former step, using the interrelationship between scenario variables and spatial states to first assign a quantitative development scale and constrain a spatial location and then to quantify and spatialize the construction conditions of different scenarios to obtain the spatial growth layout of the town under different development scenarios for achieving the sustainable development goal of a synergistic co-existence between ecosystems and social systems. In addition, the UGB has both rigid and flexible characteristics. The rigid boundary is an insurmountable ecological bottom line for urban built-up land, which is permanent and does not change with urban expansion; the flexible boundary is a dynamic boundary in different periods from the perspective of land demand for social development, which is time-sensitive and can be adjusted appropriately according to the urbanization level.

#### 4.2.2. Establishment of Supporting Policies for Multi-Party Coordination

**Figure 8:**
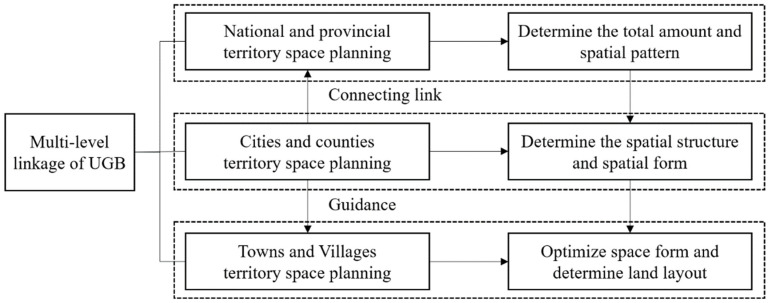
Multilevel planning framework of China’s urban development boundary system.

In China, the study of UGBs is still in the exploratory stage, and the lack of targeted supporting management mechanisms has greatly reduced their practicability. The operation of the UGB system involves the interests of the government, the market, and the public. In the context of land and space reform, it is necessary to improve the supporting policy guarantee system at four different levels: national legislation, government organization, market regulation and control, and public participation. First, the national legislation should clarify the legal status of UGBs. Priority can be given to selecting cities with the conditions necessary to carry out local pilot legislation, and then national laws and regulations can gradually be formulated and introduced. Second, the government should improve the protection system of spatial planning. The government should organize itself from “strictly controlling the approval process, strengthening implementation supervision, and implementing accountability”. Third, from the prospective of market regulation, financial support for land development should be guaranteed. The renewal of the stock of land can be encouraged by means of plot ratio encouragement and financial compensation policies to encourage market participation, and new construction land can be encouraged by means of taxation. Finally, public participation needs to be strengthened through feedback on the implementation process. Public participation can be strengthened through the opening of planning public platforms, the promotion of WeChat public numbers, and the organization of hearings, so that non-profit organizations can be informed of urban construction and development information in an open and transparent manner, and a feedback mechanism for monitoring decisions can be established. In this way, the city will be able to promote the fair, scientific, and modern development of territorial spatial governance. In addition, corresponding to the “five levels and three types” system of territorial spatial planning in China presently, it is recommended that a multi-level transmission mechanism of “national–provincial–city–county–township” be set up. The boundaries of the planning system at different levels should also have different responsibilities and tasks (Figure 8).

#### 4.2.3. Improving the Adjustment System for Implementing Evaluations

**Figure 9:**
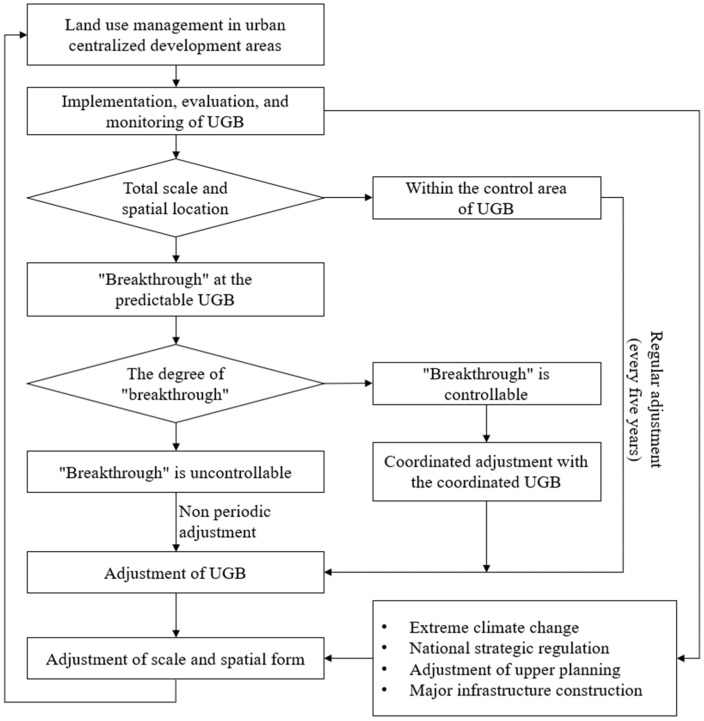
Assumption of dynamic adjustment process of “regular + irregular” UGB.

At this stage, it is difficult to assess the effects of UGB implementation in a short period of time, as there are few cases of UGB in China, and the practice period is relatively short. Few studies in China have focused on the control effects of land conversion inside and outside the boundary and ecological environment optimization after the implementation of UGB. The consistency evaluation guideline is an international indicator system that is commonly used to evaluate the effectiveness of UGB implementation by comparing the spatial match between the current site and the planned layout. Future research can consider the UGB as a multi-objective public policy and adopt four levels of evaluation criteria including efficiency, effectiveness, responsiveness, and equity. In addition, the “six-year regular revision and out-of-cycle adjustment” model of Portland was used as a reference, and a dual insurance model of “regular adjustment with non-regular adjustment” was proposed. Regular adjustment is also known as proactive adjustment, such as Beijing’s regular mechanism of “annual physical examination and five-year evaluation”, in which the land use scale and space inside and outside the boundary are adjusted appropriately according to the evaluation results and the prescribed procedures, considering the major strategic needs of the country and adjustment of the superior plan, etc. Irregular adjustment is also called passive adjustment, that is, when the boundary is breached, it needs to be adjusted passively. When the scale index is breached, the adjustment procedure of national spatial planning should be initiated. When the spatial location is breached because of major disasters or changes in the external environment, strict adjustment and approval procedures should be followed, and land use in other areas should be reduced accordingly for adjustment (Figure 9).

## 5. Conclusions

This study presents a visual analysis of the research studies on UGB worldwide based on the bibliometric software CiteSpace, focusing on their development history, delineation methods, and supporting policies in detail, and we propose reference suggestions for UGB’s subsequent practice in China. The results show that: (1) UGB theory was first proposed by American cities to cope with urban sprawl, and it has become one of the useful tools in guiding smart urban growth around the world. Theoretical and practical research have been developed in foreign countries. Meanwhile, in China, the theoretical research has focused on the conceptual connotation and Chinese and foreign comparison and delineation methods. Few practical studies have addressed the delineation of standards, technical processes, and management implementation in China. (2) UGB delineation methods can be divided into qualitative and quantitative studies, and quantitative delineation methods can be divided into the reverse restriction method based on ecological protection and the forward expansion method based on social development needs. The reverse restriction method is based on ecological sensitivity assessment, land suitability, and resource capacity evaluation, and the forward expansion method is based on urban growth simulation based on the CA optimization model. (3) Foreign countries have various and flexible policies to support the implementation of UGB, such as taxation policies, zoning policies, and public transportation-oriented policies, whereas China has not yet formed a system and lacks operational experience. Meanwhile, the implementation effect of UGB in foreign countries has regional differences, and there is little progress in this area of the research in China.

By fully integrating UGB with China’s national conditions and placing it under the macro-perspective of territorial spatial planning, we suggest the development of UGB in China from three aspects: delineating a coordinated UGB system for protection and development, formulating a supporting mechanism with multi-subject participation and multi-party linkage, and improving a dynamic adjustment system to cope with uncertain urban development and the evaluation of implementation effects. Future research should focus on the influencing factors and driving mechanisms of urban growth, and with both technical and policy attributes, it should explore the integrated urban growth management system of “planning, preparation, implementation, and operation”.

## Figures and Tables

**Table 1:** Supporting policy tools for urban growth boundaries in the United States.

Tool Category	Concrete Content	Purpose
Taxation policy	Setting a differential tax on urban and agricultural land inside and outside the border leads to more urban development within the border and more agricultural land development outside the border. For example, a lower tax rate is set for construction land, and a higher tax rate is set for agricultural land inside the boundary.	To direct urban expansion within the boundary and reduce and control development outside the boundary.
Zoning policy	Includes inclusive zoning (encouraging multi-type development and construction), group zoning (guiding residential development in well-equipped areas), intensity-restricted zoning (stipulating minimum development intensity), and exclusive zoning (separating agricultural and protected natural lands, etc.).	Combined with the concrete implementation of the boundary, it can effectively promote compact city and efficient land use.
Public transit-oriented development policy	Encourages the development and construction of high-density communities in the surrounding areas of public transport hubs and plans and designs a number of alternative public transport lines.	Strengthens the intensity and comprehensive benefits of urban land use and promotes intensive land use.
Policy on adequate provision of public facilities	Guides urban construction in areas with complete public infrastructure.	The effective regulation of urban development in time and space is helpful forimproving the efficiency of regional land interests.
Infrastructure improvement plan	Guides the planning of facilities for the next 5–10 years and the government to rationally arrange the financial expenditure for urban development and makes land developers aware of the future allocation of facilities.	

## Data Availability

The data presented in this study are available on request from the corresponding author.

## Appendix A

**Table A1:** English translation corresponding to Chinese words.

Chinese Words	English Translation	Chinese Words	English Translation
城市增长管理	Urban growth management	生态适宜性	Ecological suitability
精明增长	Smart growth	多规合一	Multi-planning Integration
绿色基础设施	Green infrastructure (GI)	城镇开发边界	Cities and towns development boundary
城市化	Urbanization	国土空间规划	Territorial spatial planning
城市增长边界	Urban growth boundary	空间管控	Space management
城市蔓延	Urban sprawl	俄勒冈州	Oregon, Portland
经济增长	Economic growth	委员会	Committee
土地管理	Land management	土地规划	Land use planning
元胞自动机	Metacellular automata	自然资源保护	Natural resource protection
城市服务边界	Urban service boundary	思想源头	Ideological source
城市总体规划	Urban master planning	土地保护	Land resource protection
城市空间增长边界	Urban spatial growth boundary	总体架构	Overall structure
新型城镇化	New urbanization	空间规划体系	Spatial planning system
城市开发边界	Urban development boundary	城市规划	Urban planning
